# AURKB promotes gastric cancer progression via activation of *CCND1* expression

**DOI:** 10.18632/aging.102684

**Published:** 2020-01-25

**Authors:** Min Nie, Yadong Wang, Zenong Yu, Xinyu Li, Yexuan Deng, Ying Wang, Dongjun Yang, Qixiang Li, Xiangwei Zeng, Junyi Ju, Ming Liu, Quan Zhao

**Affiliations:** 1 The State Key Laboratory of Pharmaceutical Biotechnology, School of Life Sciences, Nanjing University, Nanjing, China

**Keywords:** AURKB, CCND1, H3S10ph, proliferation, gastric cancer

## Abstract

Aurora kinase B (AURKB) triggers the phosphorylation of serine 10 on histone H3 (H3S10ph), which is important for chromosome condensation and cytokinesis during mitosis in mammals. However, how exactly AURKB controls cell cycle and contributes to tumorigenesis as an oncoprotein under pathological conditions remains largely unknown. Here, we report that AURKB promotes gastric cancer cell proliferation *in vitro* and *in vivo*. Silencing AURKB expression inhibits gastric cell proliferation and arrests the cell cycle in G_2_/M phase. We demonstrate that cyclin D1 (CCND1) is a direct downstream target of AURKB that plays a key role in gastric cancer cell proliferation. AURKB is able to activate the expression of CCND1 through mediating H3S10ph in the promoter of the *CCND1* gene. Furthermore, we show that AZD1152, a specific inhibitor of AURKB, can suppress the expression of CCND1 in the gastric cancer cells and inhibit cell proliferation *in vitro* and *in vivo*. Importantly, we found that high AURKB and CCND1 expression levels are correlated with shorter overall survival of gastric cancer patients. This study demonstrates that AURKB promotes gastric tumorigenesis potentially through epigenetically activating *CCND1* expression, suggesting AURKB as a promising therapeutic target in gastric cancer.

## INTRODUCTION

Gastric cancer is the fifth most common cancer and the third leading cause of cancer-related death worldwide [1]. So far, in spite of significant advances in early diagnosis and treatment including radiotherapy and chemotherapy, the overall survival rate of gastric cancer patients still remains poor, with a five-year overall survival rate of approximately 30% or less [2]. Although much effort has centered on probing the pathogenesis of the disease, the molecular mechanisms underlying the process are still largely unknown.

In mammals, there are three types of Aurora kinases: Aurora kinase A (AURKA), AURKB, and Aurora kinase C (AURKC). AURKA is mainly involved in centrosome maturation, separation and bipolar spindle assembly whereas AURKC is primarily involved in the movement of chromosomes during mitosis in mammalian cells [3, 4]. AURKB, also known as AIM-1 or Stk-5, forms a chromosomal passenger complex (CPC) with the inner centromere protein (INCENP), Survivin, Borealin/Dasra and other proteins [5]. Studies have demonstrated that the CPC plays central roles in mitosis, mediating the correction of chromosome-microtubule attachment errors, the activation of the spindle assembly checkpoint, and the regulation of chromosome segregation and cytokinesis [5]. Aurora kinase B (AURKB) is localized on the centromeres from prophase through the metaphase-anaphase transition which mainly involved in cell division from G_2_ phase to M phase [3, 4]. Previous studies indicate that AURKB can phosphorylate histone H3 on serine 10 (H3S10) and serine 28 (H3S28), which associates with chromosome number stability and chromatin condensation during mitosis [4].

In contrast to the inhibition of AURKA, which delays mitotic entry, the inhibition of AURKB directly inhibits cytokinesis and results in catastrophic mitosis [6–8]. Therefore, AURKB has become the primary target for anticancer drugs [9]. Indeed, AURKB is frequently observed highly expressed in tissues from some tumors, such as non-small cell lung cancer, breast cancer, colorectal cancer, hepatocellular carcinoma, astrocytic tumor, germ cell tumor, thyroid cancer and leukemia, and AURKB overexpression is associated with poor prognosis [4, 10–14]. Surprisingly, the role of AURKB in cancers is not fully, and well explored, although AURKB has been demonstrated to decrease the expression of p21^WAF/CIP1^, a cell cycle inhibitor, indirectly through suppressing p53 activity to facilitate cell cycle progression that antagonizes apoptosis [15]. Overexpression of AURKB may lead to the generation of aneuploid cells with malignant and aggressive phenotypes, suggesting that the abnormal expression of AURKB is associated with tumorigenesis in human cells [15]. Although much progress have been made in elucidating the importance of AURKB in tumor biology, the molecular mechanism of AURKB in gastric cancer progression, especially in modulating cell cycle progression and proliferation, remains largely unknown.

In this study, we sought to dissect the potential roles and molecular mechanism of AURKB in gastric cancer progression, especially in cell cycle progression and proliferation. We show direct functional evidence that AURKB could directly upregulate the expression of *CCND1* which encodes cyclin D1, a key allosteric activator of the cognate cyclin-dependent kinases 4/6 (CDK4/6) during the cell cycle that is vital for the initiation of DNA replication [16]. We revealed that AURKB is able to activate the expression of CCND1 through mediating H3S10ph at the promoter of the *CCND1* gene. Additionally, we also assessed the role of AURKB kinase activity in the regulation of *CCND1* transcription and related mechanism in promoting gastric cancer cell cycle progression and proliferation. These studies not only broaden our view of the impact of AURKB-CCND1 in controlling cancer cell cycle progression and proliferation, but also raise the possibility that targeting AURKB-CCND1 axis may be a promising strategy for treatment of gastric cancer.

## RESULTS

### AURKB promotes gastric cancer cell proliferation *in vitro*

**Figure 1:**
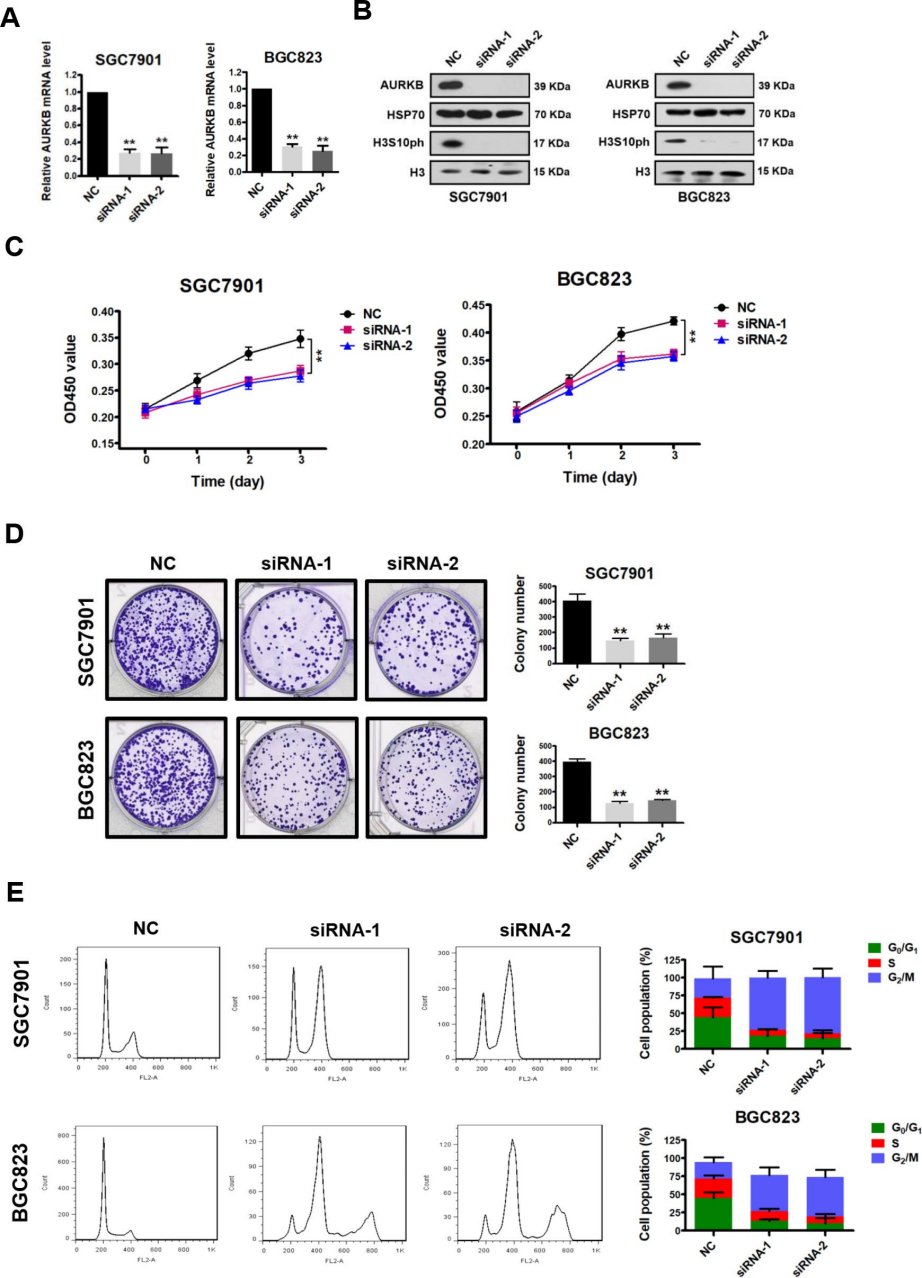
**AURKB knockdown inhibits gastric cancer cell proliferation.** (**A**) Quantitative real-time PCR analysis of the effect of AURKB knockdown by siRNA on the mRNA levels of AURKB in SGC7901 and BGC823 cells. The results shown are the means ± SDs of three independent experiments; **, P < 0.01 compared with the scrambled negative control (NC). (**B**) Western blot analysis of AURKB and H3S10ph expression in siRNA- and NC-transfected SGC7901 and BGC823 cells. HSP70 and histone H3 were used as loading controls. (**C**) CCK-8 assays showing that AURKB knockdown by siRNA significantly inhibited the proliferation of SGC7901 and BGC823 cells. The results shown are the means ± SDs of three independent experiments; **, P < 0.01 compared with the negative control. (**D**) Effects of AURKB knockdown by siRNA on the colony formation ability of SGC7901 and BGC823 cells. Left panel, representative images from colony formation assays. Right panel, the number of colonies formed by the indicated cells was quantified. Data are presented as the means ± SDs; **, P<0.01 compared with the negative control. (**E**) Flow cytometry analysis showing the cell cycle distribution of SGC7901 and BGC823 cells transfected with negative control (NC) or AURKB siRNA. Bar graphs showing the percentages of SGC7901 and BGC823 cells in the G_0_/G_1_, S and G_2_/M phases when treated with negative control (NC) or AURKB siRNAs (right panel). Each histogram bar represents the mean ± SD of three independent experiments.

AURKB, a key regulator of chromosome segregation during mitosis, plays important roles in controlling cell proliferation. To assess the role of AURKB in gastric cancer development, we first examined its effect on cancer cell proliferation *in vitro*. We knocked down AURKB in two gastric cancer cell lines (AURKB-KD), SGC7901 and BGC823, by RNAi, which reduced AURKB expression to less than 70% of that in cells transfected with the scrambled negative control (NC) (Figure 1A and 1B). Because AURKB mediates the phosphorylation of histone H3 on serine 10 (H3S10ph) [3], the levels of H3S10ph were significantly lower in AURKB-KD cells than in the scrambled control cells (Figure 1B). Then, we measured the effect of AURKB on the proliferation of gastric cancer SGC7901 and BGC823 cells using the CCK-8 kit. We found that when AURKB expression was knocked down, the proliferation of gastric cancer cells was significantly slower than that of scrambled control cells (Figure 1C). In addition, knockdown of AURKB significantly reduced the number of cell colonies formed after culture compared with that formed by NC cells (Figure 1D). Later, we performed flow cytometry analysis of the cellular DNA content in both SGC7901 and BGC823 cells to assess the effect of AURKB on cell cycle progression. We observed more AURKB-KD cells than scrambled control cells in G_2_/M phase and, correspondingly, fewer AURKB-KD cells in the S and G1 phases of the cell cycle (Figure 1E). Interestingly, polyploidy (DNA content ≥8N) was observed in AURKB-KD BGC823 cells, not in AURKB-KD SGC7901 cells. Altogether, our results indicated that knockdown of AURKB induces G_2_/M cell cycle arrest in gastric cancer cells. Thus, these results demonstrate that AURKB knockdown inhibits gastric cancer cell proliferation *in vitro*, leading to cell cycle arrest in G_2_/M phase.

### *CCND1* is a direct target of AURKB

**Figure 2:**
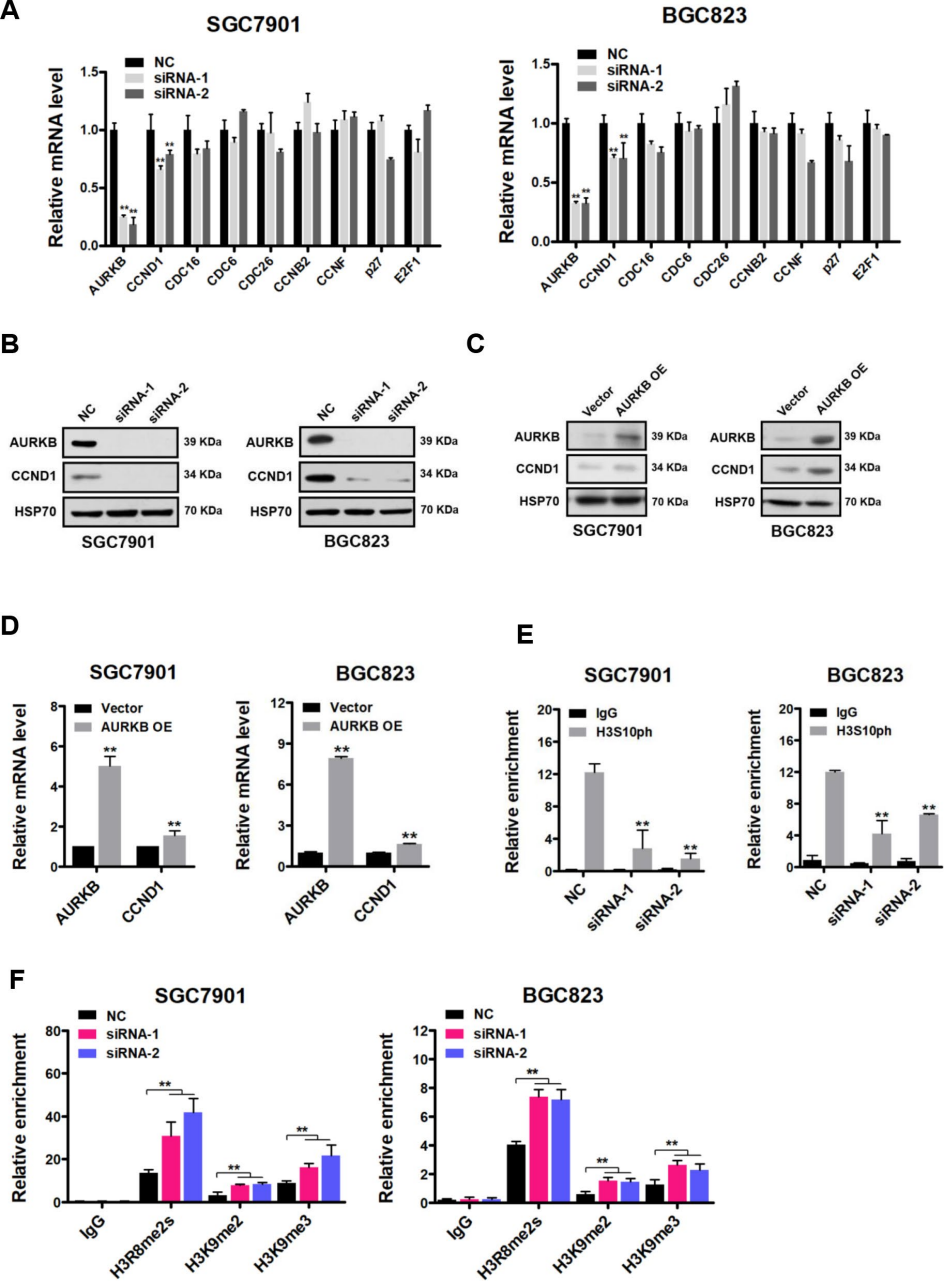
**CCND1 is a direct target of AURKB.** (**A**) Quantitative real-time PCR analysis of the effect of AURKB knockdown by siRNA on the mRNA levels of CCND1, CDC16, CDC6, CDC26, CCNB2, CCNF, p27 and E2F1 in SGC7901 and BGC823 cells relative to those in the negative control (NC) cells. The results shown are the means ± SDs of three independent experiments; **, P < 0.01 compared with the negative control. (**B**) Western blot analysis showing the effect of AURKB knockdown by siRNA on the expression of CCND1 in SGC7901 and BGC823 cells. HSP70 was the loading control. (**C**) Western blot analysis showing the effect of AURKB overexpression on the expression of *CCND1* in SGC7901 and BGC823 cells. HSP70 was the loading control. (**D**) Quantitative real-time PCR analysis of the effect of AURKB overexpression on the mRNA levels of CCND1 in SGC7901 and BGC823 cells. The results shown are the means ± SDs of three independent experiments; **, P < 0.01 compared with the negative control. (**E**–**F**) Chromatin immunoprecipitation assays showing the effect of AURKB knockdown on H3S10ph (**E**) H3R8me2s, H3K9me2, or H3K9me3 (**F**) enrichment in the *CCND1* promoter in SGC7901 and BGC823 cells. Normalized inputs of SGC7901 and BGC823 chromatin DNA were pulled down by antibodies against H3S10ph or negative immunoglobulin G (IgG). The results shown are the means ± SDs of three independent experiments; **, P < 0.01 compared with the negative control.

To understand the mechanism underlying the cell cycle arrest of gastric cancer cells induced by knocking down AURKB, we next examined the effect of AURKB on various key cell cycle regulatory molecules, including CCND1, CDC16, CDC6, CDC26, CCNB2, CCNF, p27 and E2F1, in gastric cancer cells [16]. Quantitative real-time PCR demonstrated that the expression level of *CCND1* was most consistently decreased in AURKB-KD cells compared with that in scrambled cells, whereas no significant changes in the expression of the rest of these molecules were observed (Figure 2A). The effect of AURKB on CCND1 expression was further confirmed to be significant at the protein level by western blotting (Figure 2B). These results suggest that AURKB may act to activate CCND1 expression. To further confirm this hypothesis, we subsequently established AURKB-overexpressing stable gastric cancer SGC7901 and BGC823 cell lines (AURKB-OE). We determined both the mRNA and protein levels of CCND1 in these lines using quantitative real-time PCR and western blotting, respectively. In agreement with the results of the AURKB knockdown experiment, enforced AURKB expression significantly increased both the mRNA and protein levels of CCND1 relative to those levels in vector control cells (Figure 2C and 2D). These results indicate that AURKB positively regulates *CCND1* gene expression.

AURKB triggers the phosphorylation of histone H3 on serine 10 (H3S10ph). Thus, to examine whether AURKB directly regulates *CCND1*, we performed ChIP assays using antibodies against H3S10ph (rabbit IgG was used as control) in the *CCND1* promoter. Real-time PCR assay was performed to detect the precipitated DNA by H3S10ph antibody in the promoter of *CCND1* upon AURKB knockdown. We showed that the enrichment of H3S10ph in the gene promoter of *CCND1* was indeed markedly lower when AURKB was knocked down in gastric cancer cells than in scrambled control cells (Figure 2E). Given that H3S10 phosphorylation is generally considered to be associated with the activation of gene expression [3, 4], these results are consistent with the active role of AURKB in the regulation of *CCND1* gene expression. Furthermore, real-time PCR assay was performed to detect the precipitated DNA by H3R8me2s, H3K9me2, and H3K9me3 antibodies in the promoter of *CCND1* upon AURKB knockdown. Interestingly, we observed an increase in the enrichment of the histone marks H3R8me2s, H3K9me2, and H3K9me3 in the promoter of *CCND1* upon AURKB knockdown, indicating crosstalk between these marks and H3S10ph and enhancement of *CCND1* gene repression [17, 18].

**Figure 3:**
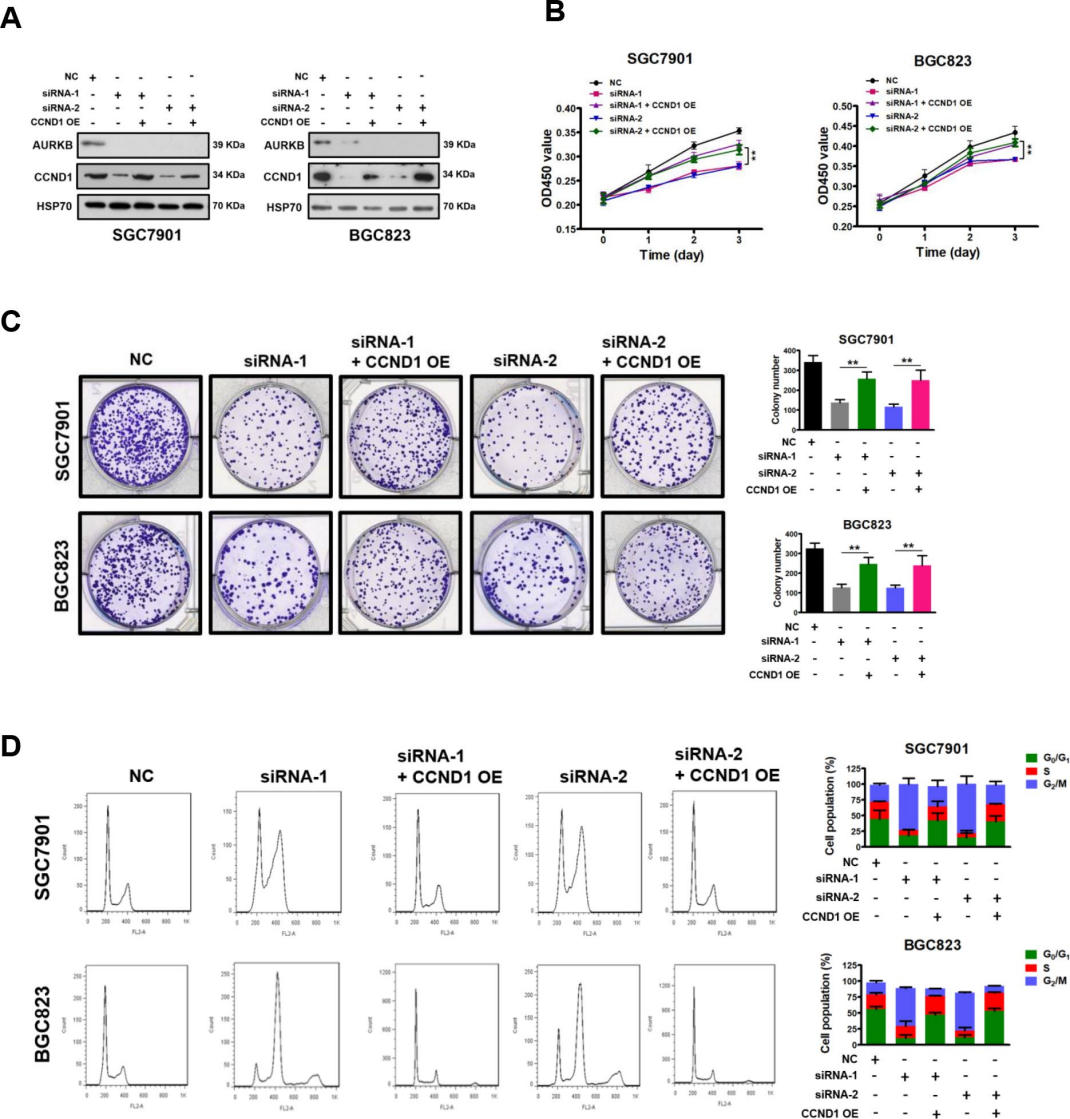
**Restoration of CCND1 abrogated the inhibition of cell growth mediated by AURKB-siRNAs.** (**A**) Western blot showing the restoration of CCND1 expression in SGC7901 and BGC823 cells transfected with AURKB siRNA or negative control siRNA. HSP70 was the loading control. (**B**) Enforced expression of CCND1 significantly abrogated the inhibition of proliferation mediated by AURKB siRNA in SGC7901 and BGC823 cells, as evidenced by CCK-8 assays. The results shown are the means ± SDs of three independent experiments; **, P < 0.01 compared with the AURKB knockdown groups. (**C**) Enforced expression of CCND1 significantly abrogated the inhibition of colony formation mediated by AURKB siRNA in SGC7901 and BGC823 cells. Left panel, representative images from colony formation assays. Right panel, the number of colonies formed by the indicated cells was quantified. Data are presented as the means ± SDs; **, P<0.01 compared with the AURKB knockdown groups. (**D**) Enforced expression of CCND1 significantly abrogated the cell cycle arrest in G_2_/M phase mediated by AURKB siRNA in SGC7901 and BGC823 cells. Right panel, bar graphs showing the percentages of SGC7901 and BGC823 cells in the G_0_/G_1_, S and G_2_/M phases. Each histogram bar represents the mean ± SD of three independent experiments.

To verify that *CCND1* is a downstream target of AURKB, we investigated whether the restoration of CCND1 expression could reverse the AURKB knockdown-mediated inhibition of gastric cancer cell proliferation. The CCND1 and AURKB protein levels were examined with western blot analyses (Figure 3A). We found that overexpression of CCND1 in AURKB knockdown SGC7901 and BGC823 cells mostly abrogated the AURKB-KD-mediated suppression of cell proliferation, reduction of cell colony formation in culture, and cell cycle arrest in G_2_/M phase (Figure 3B–3D). It is interesting to note that polyploidy (DNA content ≥8N) observed in AURKB-KD BGC823 cells almost disapeared upon overexpression of CCND1 (Figure 3D). These results confirmed that AURKB promotes gastric cancer cell proliferation by targeting *CCND1*.

### AURKB promotes gastric cancer cell growth by activating *CCND1* expression *in vivo*

**Figure 4:**
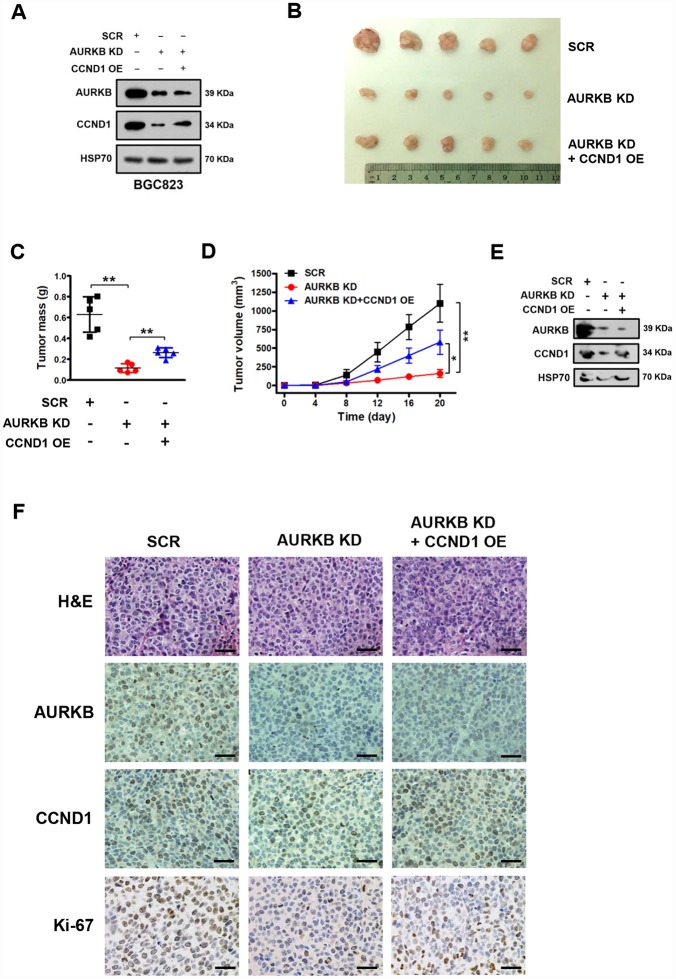
**AURKB promotes gastric cancer cell growth by regulating CCND1 *in vivo*.** (**A**) Western blot analysis of the protein levels of AURKB, CCND1 and HSP70 in stable BGC823 cell lines. HSP70 was used as the endogenous loading control. (**B**) Photograph of xenograft tumors excised from mice in the scrambled (SCR) control, AURKB KD and AURKB KD + CCND1 OE groups. (**C**) Tumor masses of xenograft tumors excised from mice in the SCR control, AURKB KD and AURKB KD + CCND1 OE groups. Data are presented as the means ± SDs; **, P < 0.01. (**D**) Tumor volumes were examined on the indicated days. Data are presented as the means ± SDs; *, P < 0.05, **, P < 0.01. (**E**) Western blotting analysis of AURKB and CCND1 expression in xenograft tumors excised from mice in the SCR control, AURKB KD and AURKB KD + CCND1 OE groups. HSP70 was used as the endogenous loading control. (**F**) Hematoxylin and eosin (H&E) staining and IHC staining of AURKB, CCND1 and Ki-67 in xenograft tumors excised from mice in the SCR control, AURKB KD and AURKB KD + CCND1 OE groups.

Based on the above results, we demonstrated that AURKB can promote the proliferation of gastric cancer cells by regulating the expression of CCND1 *in vitro*. Next, we sought to explore whether AURKB could promote gastric cancer cell proliferation *in vivo*. We successfully established stable BGC823 gastric cancer cell lines with AURKB knockdown and with AURKB knockdown plus CCND1 overexpression (Figure 4A). Later, these cells were subcutaneously injected into the flank of nude mice. Twenty days later, the nude mice were sacrificed, and tumor tissues were collected for analysis. We found that the silencing of AURKB significantly inhibited xenograft growth, while the overexpression of CCND1 readily reversed the inhibition of cell growth mediated by AURKB knockdown *in vivo* (Figure 4B–4D). We confirmed the changes in the protein levels of AURKB and CCND1 in the xenograft tissues. The western blotting results showed that the protein levels of AURKB and CCND1 were significantly lower in the AURKB knockdown group than in the SCR control group, whereas CCND1 expression was increased in tumors with CCND1 overexpression (Figure 4E). We obtained consistent results from the tissue immunohistochemical staining analyses with antibodies against AURKB, CCND1 and Ki-67 (Figure 4F). These results reveal that AURKB can promote the growth of gastric cancer cells through activating the expression of CCND1 *in vivo*.

### Inhibition of AURKB kinase activity represses *CCND1* expression in gastric cancer cells

**Figure 5:**
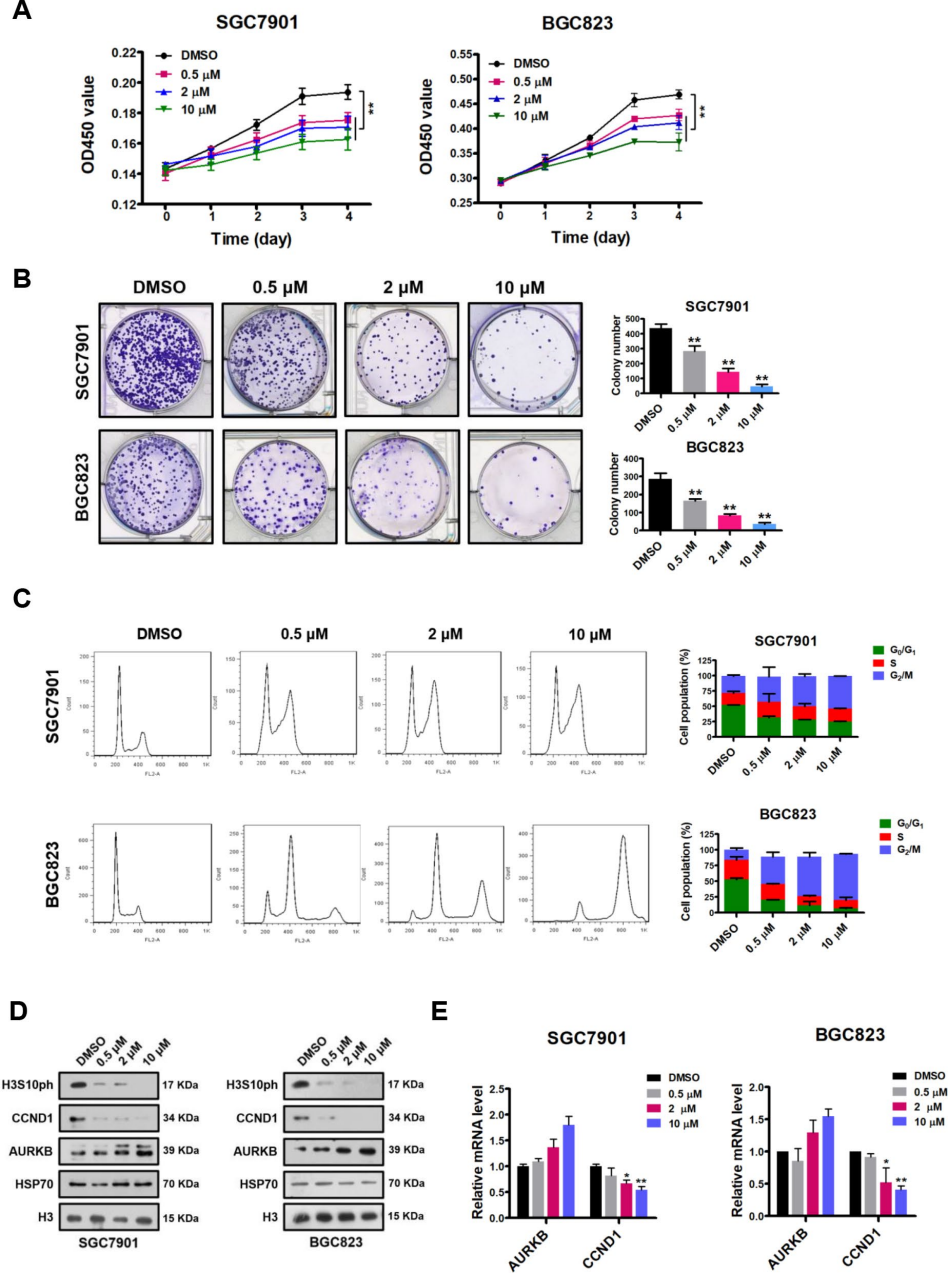
**AZD1152 suppresses cell proliferation and represses the expression of CCND1 by inhibiting the enzymatic activity of AURKB in gastric cancer cells.** (**A**) CCK-8 assays showing the effect of different concentrations of AZD1152 (0.5 μM, 2 μM and 10 μM; DMSO as the control) on the proliferation of SGC7901 and BGC823 cells. The results shown are the means ± SDs of three independent experiments; **, P < 0.01 compared with the DMSO control. (**B**) Colony formation assays showing the effects of different concentrations of AZD1152 (0.5 μM, 2 μM and 10 μM; DMSO as the control) on the colony formation ability of SGC7901 and BGC823 cells. Left panel, representative images from colony formation assays. Right panel, the number of colonies formed by the indicated cells was quantified. Data are presented as the means ± SDs; **, P<0.01 compared with the DMSO control. (**C**) Flow cytometry analysis showing the effect of different concentrations of AZD1152 (0.5 μM, 2 μM and 10 μM; DMSO as the control) on the cell cycle distribution. Bar graphs showing the percentages of SGC7901 and BGC823 cells in the G_0_/G_1_, S and G_2_/M phases when treated with different concentrations of AZD1152 (right panel). Each histogram bar represents the mean ± SD of three independent experiments. (**D**) Western blot analysis of the protein levels of AURKB, CCND1 and H3S10ph in SGC7901 and BGC823 cells treated with different concentrations of AZD1152 (0.5 μM, 2 μM and 10 μM; DMSO as the control). HSP70 and histone H3 were used as the endogenous loading controls. (**E**) Quantitative real-time PCR analysis of AURKB and CCND1 expression in SGC7901 and BGC823 cells treated with different concentrations of AZD1152 (0.5 μM, 2 μM and 10 μM; DMSO as the control); *, P < 0.05; **, P < 0.01. The results shown are the means ± SDs of three independent experiments. GAPDH was used as the endogenous control for mRNA expression analysis.

Since AURKB is a key regulator during cell mitosis and is highly expressed in rapidly proliferating cells, it is considered a promising target for the development of inhibitors for cancer therapy [3]. In addition, the results obtained above strongly suggest that AURKB plays a proliferation-promoting role in gastric cancer cells. Given that there is a selective specific Aurora B kinase inhibitor available—AZD1152 (barasertib), which is currently in the clinical phase II stage for the inhibition of the proliferation of various cancers, including leukemia and some solid tumors [4, 9]—we assessed the inhibitory effect of this inhibitor on gastric cancer cells. Thus, we treated gastric cancer SGC7901 and BGC823 cells with different concentrations of AZD1152. We found that AZD1152 significantly inhibited cell proliferation, decreased cell colony formation in culture, and led to cell cycle arrest in G_2_/M phase in BGC823 and SGC7901 cells in a dose-dependent manner (Figure 5A–5C). Similar to AURKB knockdown, polyploidy (DNA content ≥8N) was observed in AZD1152-treated BGC823 cells, not in AZD1152-treated SGC7901 cells (Figure 5C). As expected, the western blot analysis results showed that AZD1152 inhibited the enzymatic activity of AURKB, leading to significantly reduced H3S10ph levels (Figure 5D). Moreover, also as expected, the expression of CCND1 was consistently reduced, although there was a slight increase in AURKB expression (Figure 5D and 5E). These results are consistent with those obtained in the AURKB knockdown experiments, indicating that the inhibition of AURKB kinase activity results in the repression of CCND1 expression in gastric cancer cells and subsequently inhibits cell proliferation.

### Inhibition of AURKB kinase activity represses the expression of CCND1 and decreases gastric cancer growth *in vivo*

**Figure 6:**
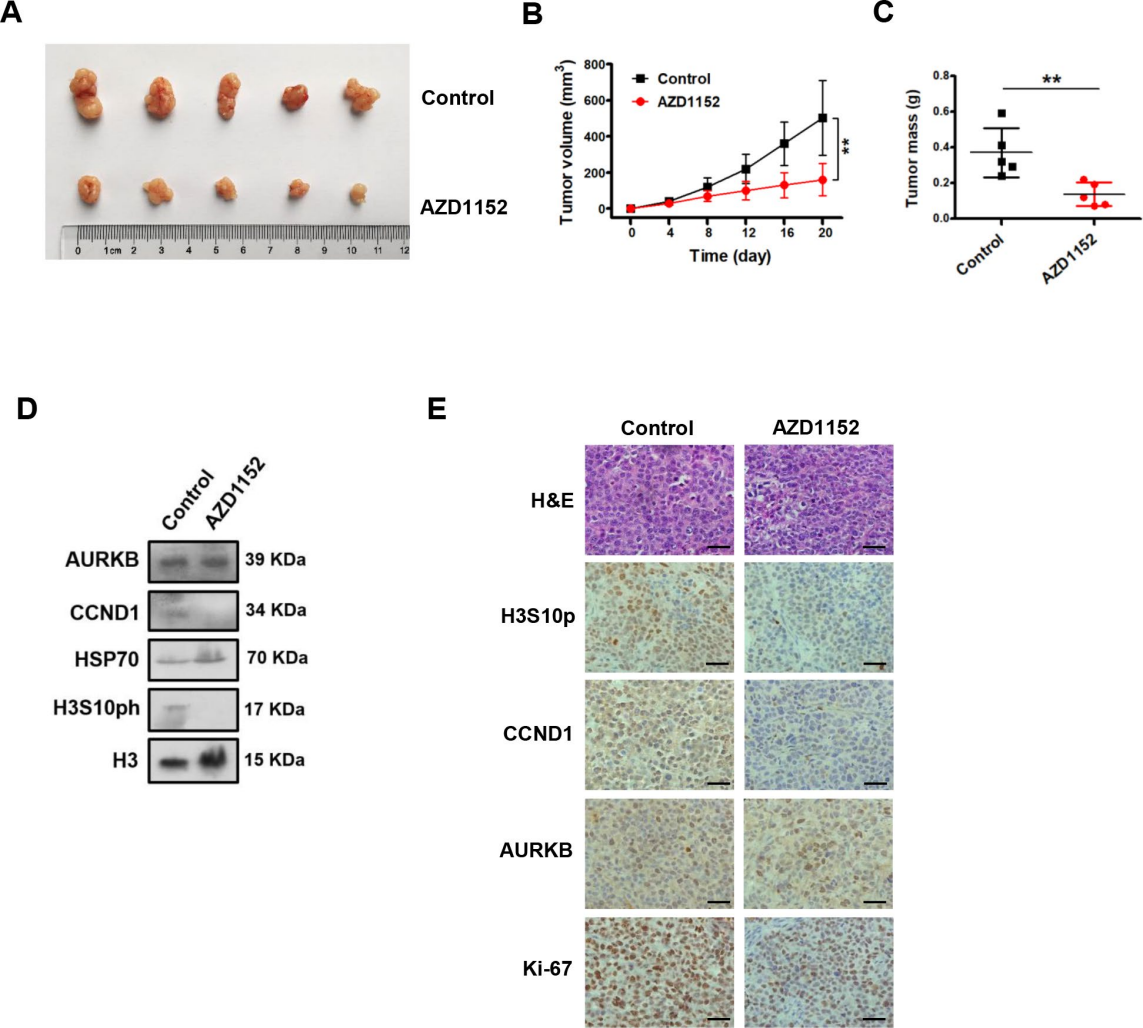
**Inhibition of AURKB kinase activity represses the expression of CCND1 and decreases gastric cancer growth *in vivo*.** (**A**) Photograph of xenograft tumors excised from mice treated with AZD1152 (25 mg/kg). (**B**) Tumor volumes were examined on the indicated days. Data are presented as the means ± SDs; **, P < 0.01. (**C**) Tumor mass of xenograft tumors excised from mice treated with AZD1152 (25 mg/kg). (**D**) Western blot analysis of AURKB, CCND1 and H3S10ph expression in xenograft tumors treated with AZD1152 (25 mg/kg). HSP70 and histone H3 were used as the endogenous controls. (**E**) Hematoxylin and eosin (H&E) staining and IHC staining of AURKB, CCND1, H3S10ph and Ki-67 in xenograft tumors excised from mice treated with AZD1152 (25 mg/kg) and control.

We showed that the inhibition of AURKB kinase activity by AZD1152 repressed CCND1 expression in gastric cancer cells and decreased cell proliferation *in vitro*. We then tested whether the inhibition of AURKB kinase activity by AZD1152 could reduce gastric cancer growth *in vivo*. Thus, BGC823 cells were subcutaneously injected into the flank of nude mice. Established subcutaneous xenografts of BGC823 cells were intraperitoneally treated with AZD1152 (25 mg/kg) or control buffer every two days. Twenty days later, the nude mice were sacrificed, and tumor tissues were collected for analysis. We found that the inhibition of AURKB significantly inhibited the growth of xenograft tumors (Figure 6A–6C).

Subsequently, we assessed whether the inhibition AURKB kinase activity corresponded to decreased levels of CCND1 *in vivo*. We examined the protein levels of CCND1 in the xenograft tissues (Figure 6D) by Western blotting assays. As expected, the protein levels CCND1 were noticeably decreased in the AZD1152-treated xenograft tissues. Meanwhile, the protein levels of histone H3S10ph was also significantly reduced in the AZD1152-treated group relative to those in the control group, without any change in the AURKB protein level. We showed consistent results in tissue immunohistochemical staining analyses with antibodies against histone H3S10ph, CCND1, AURKB and Ki-67 (a proliferative marker) (Figure 6E). Therefore, these results confirm that the inhibition of AURKB kinase activity represses the expression of CCND1 and decreases gastric cancer growth *in vivo*.

### Expression of AURKB and CCND1 is upregulated in gastric cancer tissues, and these expression levels are associated with poor prognosis in gastric cancer patients

**Figure 7:**
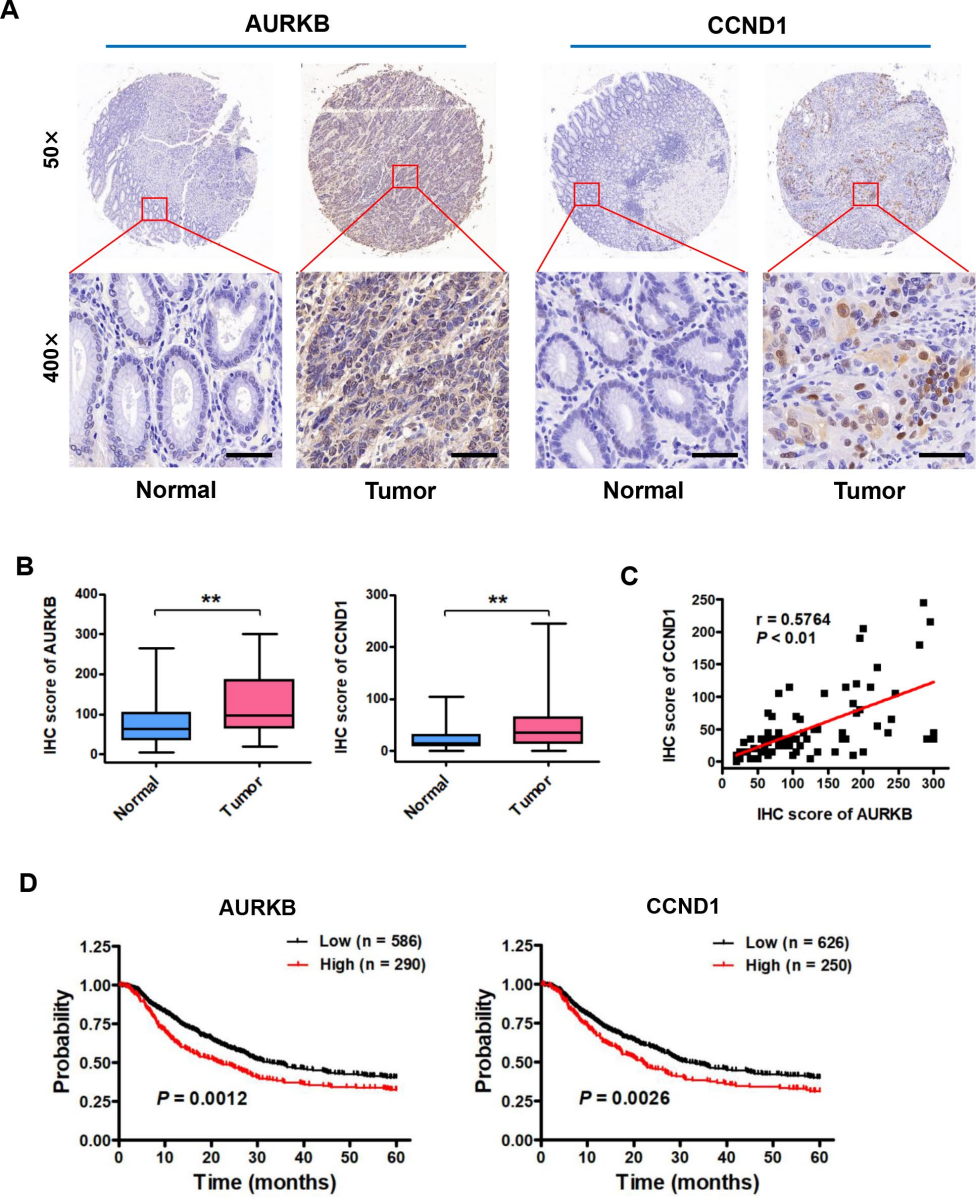
**Expression of AURKB and CCND1 is upregulated in gastric cancer tissues, and these expression levels are associated with poor prognosis in gastric cancer patients.** (**A**) Representative images of immunohistochemical (IHC) staining of AURKB and CCND1 in human gastric cancer tissues and matched normal tissues (n = 80). (**B**) Total IHC score for AURKB and CCND1 in human gastric cancer tissues (Tumor) and matched normal tissues (Normal). **P < 0.01 compared with the matched normal tissue control. (**C**) The correlation between AURKB and CCND1 expression was evaluated using Pearson correlation analysis (n = 80; r = 0.5764; P < 0.01). (**D**) Analysis of data from the Kaplan-Meier plotter database suggested that high expression levels of AURKB (left panel; P = 0.0012) and CCND1 (right panel; P = 0.0026) are negatively correlated with 5-year overall survival in gastric cancer patients.

AURKB, a conserved serine/threonine kinase, plays important roles in the process of cell mitosis through associating with microtubules. However, thus far, there are few reports about AURKB expression and function in human gastric cancer. To investigate the clinical significance of AURKB expression in gastric cancer patients, we examined the expression of AURKB by immunohistochemical (IHC) staining on a set of human tissue arrays containing 80 gastric cancer samples and adjacent normal gastric tissue samples as controls. IHC staining of AURKB or CCND1 in the tissue was scored according to the semiquantitative H score independently by two pathologists blinded to the clinical data, which takes into account both the intensity of the color reaction and the percentage of positive cells. We found that AURKB expression was significantly upregulated in cancer tissues compared with that in normal gastric tissues (Figure 7A and 7B). We obtained similar results from CCND1 staining in those samples (Figure 7A and 7B). Interestingly, the expression levels of AURKB and CCND1 were significantly positively correlated (Figure 7C). For survival analyses, patient overall survival times stratified by the expression of the gene of interest were presented as Kaplan-Meier plots and tested for significance using log-rank tests from the Kaplan-Meier plotter database. The details of AURKB are as follows: Affy ID: 209464_at (AURKB), cutoff value used in analysis was 303; high n = 290, low n = 586. The details of CCND1 are as follows: Affy ID: 208712_at (CCND1), cutoff value used in analysis was 1539; high n = 250, low n = 626. Importantly, Kaplan-Meier survival analysis showed that gastric cancer patients with high AURKB and CCND1 expression levels had shorter overall survival than those with low AURKB and CCND1 expression levels (Figure 7D). These results indicate that the expression of AURKB and CCND1 is upregulated in human gastric cancer tissues and is correlated with poor prognosis in gastric cancer.

## DISCUSSION

Although the first Aurora kinase was discovered in 1995 and AURKB has been implicated in the tumorigenesis of various cancers [9, 19], the direct links are not fully understood [4, 15]. In this study, we defined the biological effects of AURKB and the potential underlying mechanism in gastric cancer both *in vitro* and *in vivo*. We demonstrated that the proliferation of SGC7901 and BGC823 cells was significantly reduced *in vitro* and *in vivo* when AURKB expression was knocked down. Notably, we firstly identified *CCND1* as a direct downstream target of AURKB that plays a key role in gastric cancer cell cycle and proliferation.

CCND1, also known as BCL1/PRAD1, is generally highly expressed in human tumors [16]. Aberrant expression of CCND1 in the early stage of malignancy can lead to chromosomal rearrangements and aberrant gene amplification, suggesting that CCND1 might play a crucial role in carcinogenesis [20–22]. CCND1 forms active complexes with the cyclin-dependent kinases 4/6 (CDK4/6) to phosphorylate retinoblastoma protein (pRb) to drive progression from G1 phase to S phase [16]. It has been shown that *CCND1* promotes tumor progression mainly by shortening G_1_ phase, resulting in an increase in the number of cells progressing through G1 phase [23]. Our study showed that AURKB knockdown significantly reduced the number of cells in the G1 and S phases and arrested cells in G_2_/M phase. Furthermore, compensation experiments in which CCND1 expression was enforced in AURKB knockdown cells showed complete recovery of cells in the G1 and S phases during the cell cycle. These results clearly indicate that *CCND1* is a key downstream target of AURKB controlling the cell cycle and further support the tumor-promoting role of CCND1 in tumorigenesis.

Another important significance of this study is that phosphorylation of histone H3 on Ser 10 (H3S10ph), an important histone posttranslational modification mark triggered by AURKB, could mediate the activation of *CCND1* gene expression. We obtained consistent *in vitro* and *in vivo* results when we utilized AZD1152 to inhibit the kinase activity of AURKB. H3S10ph has been linked to various cellular processes, such as chromosome condensation and cytokinesis, apoptosis and DNA damage repair, and gene expression regulation [24]. In mammalian cells, this modification initiates the early condensation of heterochromatin surrounding the centromeres during late G2 phase [25]. Starting from prophase, this phosphorylation spreads along the chromosomes and is maintained until anaphase, when dephosphorylation occurs [26, 27]. Thus, histone H3S10ph is regarded as a marker of mitosis due to this unique pattern [3, 5]. During mitosis, H3S10ph acts in concert with H3K14ac to induce the dissociation of the heterochromatin protein HP1 from chromatin, which cooperates with the histone methyltransferase SUV39h to trigger histone H3K9me2/3 modifications in pericentric heterochromatin [28–31]. A recent work showed that AURKB can also phosphorylate Ser 121 of histone H2AX to promote its autophosphorylation and activation [32]. In addition to histone substrates, AURKB can phosphorylate centrosome protein A (CENP-A), Hec1, mitotic kinesin-like protein 1 (MKLP1), RacGAP1, Kif2A, and class IIa HDACs to maintain the stabilization of the central spindle and to promote cytokinesis [5, 33–36]. However, during interphase, H3S10ph can be related to enhanced transcription, likely to support an open chromatin structure [37–42]. It has been shown that in Drosophila, H3S10ph is required for the recruitment of positive transcription elongation factor b (P-TEFb) on heat shock genes [40]. In mammals, H3S10ph can enhance the recruitment of GCN5, leading to acetylation of K14 on the same histone tail [43, 44]. In response to serum, at the *c-Jun* and *c-Fos* genes, H3S10ph facilitates the recruitment of 14-3-3 proteins, whose binding affinities increase when H3K9ac or H3K14ac occurs simultaneously [45, 46]. Similarly, at the *FOSL1* enhancer, H3S10ph can crosstalk with H4K16ac to generate a nucleosome platform for the recruitment of BRD4 and P-TEFb to activate transcriptional elongation in response to serum stimulation [47].

In this study, we found that AURKB-mediated H3S10ph can be enriched at the *CCND1* gene promoter to activate gene expression prior to G_2_/M phase. Correspondingly, we found that the enrichment of H3K9me2/3 and H3R8me2s on the *CCND1* promoter was significantly increased when AURKB was knocked down. These results suggest that the histone mark H3S10ph may inhibit the methylation of histones H3K9 and H3R8 or, alternatively, may block the binding of H3K9me2/3-binding proteins, e.g., HP1 or H3R8me2s-mediating protein, PRMT5 [17, 18]. This finding is in agreement with the events at the MMTV promoter in response to steroid hormones, where induced H3S10ph results in the displacement of HP1γ and the recruitment of the ATP-dependent remodeling complex to induce transcriptional activation [48]. However, it is likely that the *CCND1* promoter structure is conducive to H3S10ph-induced gene activation or that the gene setting might need preparation for subsequent cell cycle programming before M phase [5, 41]. In fact, histone H3S10ph has been found during S phase for the first two cell divisions in embryogenesis [49]. Recently, H3S10ph has been revealed to promote the expression of noncoding minor satellite RNA (cenRNA) in ESCs during S phase to regulate telomerase activity [50].

Over the past several years, AZD1152, a specific inhibitor of AURKB, has attracted great attention from researchers [51, 52]. AZDl152, also known as barasertib, has a 3000-fold greater selectivity for AURKB than for AURKA [52]. AZD1152 inhibited cell growth *in vivo* in a dose-dependent manner in many cancers, including leukemia, colorectal cancer, breast cancer, lung cancer, and some other solid tumors, and it is in the clinical phase II stage [52–55]. We observed that AZD1152 inhibited gastric cancer cell proliferation both *in vitro* and *in vivo*. The induction of polyploidy (DNA content ≥8N) is the hallmark of cell phenotypic changes by AURKB inhibition [54, 56]. However, our study showed that the cancer cell line SGC7901 behaved differently than the BGC823 cell line when treated with AZD1152, remaining in a tetraploid state and not reentering S phase, thus reversing endoreduplication. We expect that amplification or increased expression of c-myc in SGC7901 cells might result in this dissimilar response to AZD1152 [54, 56]. However, the expression of CCND1 was decreased in a dose-dependent manner upon AZD1152 treatment, in agreement with its inhibitory anticancer effect on cell proliferation.

In conclusion, we showed that AURKB promotes gastric tumorigenesis by activating *CCND1* expression. We provide evidence that AURKB may play an onco-protein role during the cell cycle and proliferation. We believe that targeting AURKB-CCND1 axis will be a promising strategy for gastric cancer therapy.

## MATERIALS AND METHODS

### Cell cultures

Human gastric cancer SGC7901 (poorly differentiated and metastatic) and BGC823 (poorly differentiated) cells were grown in RPMI 1640 (Gibco Life Technologies, Paisley, UK) supplemented with 10% fetal bovine serum (FBS; Gibco Inc., Los Angeles, CA, USA) and 1% penicillin-streptomycin (Beyotime Biotechnology, China). HEK293T cells were cultured in DMEM (Gibco Life Technologies, Paisley, UK) supplemented with 10% FBS and 1% penicillin-streptomycin (Beyotime Biotechnology, China). Cells were cultured at 37°C in a humidified atmosphere of 95% air and 5% CO_2_. The human gastric cancer cell lines were recently authenticated by Genetic Testing Biotechnology Corporation (Suzhou, China) using short tandem repeat (STR) profiling. All lines were found to be negative for mycoplasma contamination.

### siRNA transfection assays, transfection and plasmids

Small interfering RNA (siRNA) sequences were commercially synthesized (GenePharma, Shanghai, China). Transient transfection was performed using Lipofectamine 2000 reagent (Invitrogen, Carlsbad, CA) when cells were 50~60% confluent, according to the manufacturer’s protocol. The sequences of AURKB siRNA-1 and siRNA-2 were 5’-GGUGAUUCACAGAGACAUA-3’ and 5’-GGAUCUACUUGAUUCUAGA-3’, respectively.

To establish stable AURKB knockdown cells, the AURKB shRNA target sequences and scrambled sequences were inserted into the pLVX-IRES-mCherry vector. HEK293T cells were grown in DMEM with 10% fetal bovine serum for retrovirus production. Then, gastric cancer BGC823 cells were transfected with the viral products from the HEK293T cells. For CCND1 overexpression, human *CCND1* cDNA was cloned into the retroviral vector MSCV-IRES-HA-GFP. AURKB knockdown BGC823 cells were transfected with the retrovirus product from the HEK293T cells. Positive stable cells were selected by fluorescence-activated cell sorting (FACS) as described previously [57].

### qRT-PCR analysis

Total RNA isolation was performed using TRIzol reagent (Invitrogen, Carlsbad, CA, USA). Reverse transcription was conducted using a commercial One Step RT-PCR kit (Vazyme Biotech Co., China), and qRT-PCR analysis was performed using FastStart Universal SYBR Green Master Mix (Roche Applied Science, Germany) in triplicate for each condition. The housekeeping gene GAPDH was used as the internal control for gene expression normalization. The sequences of all the primers used for qRT-PCR are listed as follows: AURKB: (Forward) 5’-CGCAGAGAGATCGAAATCCAG-3’ (Reverse) 5’-AGATCCTCCTCCGGTCATAAAA-3’ CCND1: (Forward) 5’-GCTGCGAAGTGGAAACCATC-3’ (Reverse) 5’-CCTCCTTCTGCACACATTTGAA-3’ CDC16: (Forward) 5’-AACAGAGACGGATGCTTCAAAA-3’ (Reverse) 5’-CCCCAGTAAAGTGGTCAAGGAT-3’ CDC26: (Forward) 5’-AACCAACACGCCTAGAGCTAA-3’ (Reverse) 5’-CTTCTGTTTCTTACGGGTCTCC-3’ CCNB2: (Forward) 5’-CCGACGGTGTCCAGTGATTT-3’ (Reverse) 5’-TGTTGTTTTGGTGGGTTGAACT-3’ CCNF: (Forward) 5’-CCCCGAAGATGTGCTCTTTCA-3’ (Reverse) 5’-GCCTTCATTGTAGAGGTAGGCT-3’ p27: (Forward) 5’-GGAGCAATGCGCAGGAATAA-3’ (Reverse) 5’-TGGGGAACCGTCTGAAACAT-3’ E2F1: (Forward) 5’-ACGCTATGAGACCTCACTGAA-3’ (Reverse) 5’-TCCTGGGTCAACCCCTCAAG-3’ GAPDH: (Forward) 5’-GAAGGTGAAGGTCGGAG-3’ (Reverse) 5’-GAAGATGGTGATGGGATTTC-3’.

### Western blotting

Cell lysates were prepared using commercial RIPA buffer (Beyotime Biotechnology, China). The extracted proteins (20~60 μg) were separated by SDS-PAGE and transferred onto a PVDF membrane (Millipore, Bedford, MA) using a semidry transfer system (Bio-Rad Laboratories, USA). After blocking with 5% nonfat milk, the PVDF membrane was incubated with primary antibodies against AURKB (Abcam; ab218339), CCND1 (Abcam; ab134175) and GAPDH (MBL; M171-3). The next day, following incubation with an appropriate secondary antibody, immunoreactions on the PVDF membrane were detected with an ECL chemiluminescence kit (Pierce Biotechnology, USA), and the membrane was then exposed to X-ray film (Kodak, Japan).

### Cell proliferation assay and colony formation assay

For the cell proliferation assay, cells were seeded (2×10^3^~4×10^3^ per well) in triplicate in 96-well plates. Cell proliferation was monitored by using a Cell Counting Kit-8 (Dojindo, Japan) according to the manufacturer’s instructions. For the colony formation assay, cells were resuspended in 2 ml of RPMI 1640 (10% FBS) and plated (500 per well) in triplicate in 6-well plates. After incubation for two weeks, colonies were fixed with 10% formaldehyde for 30 min, stained with 0.05% crystal violet (Sigma) for 30 min at room temperature, washed with running water to remove excessive dye and imaged by a scanner.

### Flow cytometry analysis

Cells were collected and fixed with 70% ethanol at -30°C for 24 h and were then stained with 5 mg/ml propidium iodide (PI; KeyGen, Nanjing, China). Flow cytometry analysis of the cell cycle was performed using a FACSCalibur (BD Biosciences, San Diego, CA). The data were analyzed using FlowJo (TreeStar, Ashland, OR, USA) and ModFit (BD Bioscience) software.

### Xenograft assay and AZD1152 treatment

AZD1152 and its active metabolite AZD1152-HQPA were obtained from Aladdin Reagent and Sigma-Aldrich, respectively. AZD1152-HQPA was used in the *in vitro* studies. AZD1152-HQPA was dissolved in DMSO, and cells were incubated with AZD1152-HQPA or DMSO vehicle control. The final concentration of DMSO used in cell culture medium was 1/2000 (v/v). For the *in vivo* studies, AZD1152 was dissolved in 3 M Tris buffer (pH 9.0) to a concentration of 5 mg/ml. A total of 5 × 10^6^ BGC823 cells (100 μl of PBS with 10% Matrigel) were implanted in the right flank of 6~8-week-old nude mice (Model Animal Research Center of Nanjing University). Palpable tumors were confirmed on day 4 following injection, and mice were randomized into treatment groups (5 mice per group) to receive AZD1152 or the control Tris buffer. Established subcutaneous xenografts of BGC823 cells were intraperitoneally treated with AZD1152 (25 mg/kg) or the control Tris buffer (3 M, pH 9.0) every two days. Tumors were measured every four days using a caliper, and tumor volume was calculated from two-dimensional tumor measurements by the following formula: Volume = 0.5 × length × width^2^. Subsequently, mice were sacrificed, and tumor tissue samples were collected and analyzed. All animal experimental procedures using animals were approved by the Institutional Animal Care and Use Committee of Nanjing University.

### Clinical samples and immunohistochemical (IHC) staining

Two tissue microarray (TMA) chips containing a total of 80 pairs of gastric cancer samples and matched adjacent normal tissues with follow-up data were obtained from Shanghai Biochip Co., Ltd., Shanghai, China. Immunohistochemical staining of tissue sections was performed as described previously [57]. Briefly, primary antibodies against AURKB, CCND1 and Ki-67 were used at a 1:100 dilution, along with horseradish peroxidase-conjugated anti-rabbit secondary antibody (Santa Cruz Biotechnology, Inc.). Signals were detected using an EnVision kit (Dako Laboratories, Carpinteria, CA). Positive staining was visualized as a brown color.

Immunohistochemical staining of AURKB or CCND1 in the tissue was scored according to the semiquantitative H score independently by two pathologists blinded to the clinical data [58], which takes into account both the intensity of the color reaction and the percentage of positive cells. Rare discordant scores were resolved by re-review of the slide and consultation between the pathologists. Category A documented the intensity of immunostaining as 0–3 (0, negative; 1, weak; 2, moderate; and 3, strong). For the correlation scatter plot of AURKB and CCND1 in human gastric cancer, the H score was calculated by summing the product of the different staining intensities as in category A above (0–3) with the percentage of positive cells, i.e., H score (0–300 scale) = 3×(% of 3+ cells) + 2×(% of 2+ cells) + 1×(% of 1+ cells). For survival analyses, patient overall survival times stratified by the expression of the gene of interest were presented as Kaplan-Meier plots and tested for significance using log-rank tests from the Kaplan-Meier plotter database (www.kmplot.com) [59]. The degree of correlation between patient expression patterns of AURKB and CCND1 was assessed via Pearson correlation analysis.

### Chromatin immunoprecipitation assay

The ChIP assay was performed as described previously [60]. In brief, cells were washed with 1×PBS and crosslinked with 1% formaldehyde (Sigma) and were then sonicated to generate DNA fragments with an average size of 200~500 bp. Soluble chromatin was then incubated with an antibody against H3S10ph (Millipore; 17-685), H3R8me2s (ABclonal; A2374), H3K9me2 (Abcam; ab1220) or H3K9me3 (Abcam; ab8898) overnight at 4°C, and immunoprecipitation was performed with Protein A/G beads. Real-time PCR was conducted using precipitated DNA as the template. The CCND1 promoter primer sequences were as follows: (forward) 5’-TCCACCTCACCCCCTAAATC-3’ (reverse) 5’-AGCCCAAAAGCCATCCCTGA-3’.

### Statistical analysis

All quantitative data are expressed as the mean ± SD. Student's t-test was performed using GraphPad Prism version 5 (GraphPad Software Inc, San Diego, CA, USA). P < 0.05 was considered as statistically significant difference.
